# Selection Signatures Reveal Candidate Genes for the Cornish Rex Breed-Specific Phenotype

**DOI:** 10.3390/genes15030368

**Published:** 2024-03-16

**Authors:** Minja Zorc, Tajda Horvat, Anja Tanšek, Tamara Ferme, Peter Dovč

**Affiliations:** University of Ljubljana, Biotechnical Faculty, Jamnikarjeva ulica 101, 1000 Ljubljana, Slovenia; minja.zorc@bf.uni-lj.si (M.Z.); tajda.horvat@gmail.com (T.H.); anja.tansek@bf.uni-lj.si (A.T.); tamara.ferme@bf.uni-lj.si (T.F.)

**Keywords:** cat, Cornish Rex, rex phenotype, selection signature, SNP array

## Abstract

Many coat color, behavioral and morphological traits are specific and fixed across cat breeds, with several variants influencing these traits being common among different breeds. In the domestic cat, rexoid mutations have been documented in several breeds. In the Cornish Rex, four bp deletion in the *LPAR6* gene has been found to cause a frame shift and a premature stop codon. In addition to the rexoid coat, Cornish Rex cats also have a characteristic head, ear shape and body type. Analysis of the selection signatures in the Cornish Rex genome revealed several regions that are under selective pressure. One of these is located in CFA B4, in the region where the *ALX1* gene is located. The *ALX1* gene in Burmese cats disrupts the cranial morphogenesis and causes brachycephaly in the heterozygous state. In our study, we confirmed the presence of a deletion in *LPAR6* in 20 Cornish Rex and in four F1 hybrids between Cornish Rex and domestic cat. However, we did not confirm the presence of the deletion in *ALX1* in Cornish Rex cats. Genome-wide selection signature analysis was performed using ROH islands and integrated haplotype score (iHS) statistics based on publicly available SNP array data of 11 Cornish Rex cats. The selection signatures were detected on chromosomes A1, A3, C2, B1, B4 and D1.

## 1. Introduction

The domestic cat is considered semi-domesticated rather than fully domesticated, as numerous populations are not completely isolated from wild cats and their reproduction, territory and diet are not strictly regulated [1,2]. The cat genome has been subjected to less intensive selective pressures compared to other domesticated species. While many domesticated mammals have been bred for specific functions, the majority of modern domestic cat breeds have only emerged in the last 150 years, selected predominantly for aesthetic external traits rather than functional traits [3]. Using whole genome sequence analysis of domestic and wild cats, genes under selection in domestic cat, influencing traits such as socialization, nutrient metabolism and coat pattern were identified [4]. Several coat color, behavior and morphological traits are specific and fixed within different cat breeds, and many of these variants are shared across breeds [5]. Some phenotypic traits within and across breeds are likely to be identical by descent [6,7].

One of the earliest phenotypes noted for a cat breed were the curly coats of the Cornish Rex and Devon Rex. A distinctive phenotype in mammals, characterized by a short, soft, wavy or curly coat with a degraded or absent guard hair is known as rex. Rex mutants are also characterized by abnormal whiskers, which are curly, short and break easily. The rex phenotype got his name from the rabbit Castor Rex, which was the first breed of this kind to be described [8]. Since then, rexoid phenotypes have also been discovered in other mammals. The genetic basis for this phenotype is heterogeneous. Different loci causing the rex phenotype have been identified in several species: rabbits [9], laboratory rodents [10], dogs [11,12], cats [13], cattle [14], horses [15] and guinea pigs [16].

In domestic cats, rexoid mutations have been documented in breeds including Cornish Rex [6], Devon Rex [17], German Rex [6], Oregon Rex [18], Ural Rex [13] and Selkirk Rex [7]. In Devon Rex and Selkirk Rex, causal mutations for curly hair are located in the *KRT71* gene [6,7]. However, in the Ural Rex, the causal deletion is located in *LIPH* gene [13] and in Cornish Rex, four base pair deletion was found in the exon 5 of the *LPAR6* gene (c.250_253_delTTTG) [6]. It causes a frame shift and premature stop codon at position 92 of the lysophosphatidic acid receptor 6, which is essential for maintaining the structural integrity of the hair shaft. Mutations in the *LPAR6* gene (previously named *P2RY5*) cause a phenotype which can range from woolly to sparse hair or complete hair loss in different human populations [19,20,21].

The *LPAR6* and *LIPH* genes are expressed in the inner root hair sheath of hair follicle, and are components of a signaling pathway that plays a crucial role in hair growth [19,20]. Inoue et al. (2011) have produced Liph protein-deficient mice with a curly coat phenotype [22]. Based on a series of expression studies in the mutant mice and in vitro analyses, they concluded that hair follicle development is regulated by *LIPH* and *LPAR6*. Epithelial cells in hair follicles express PA-PLA_1_α (encoded by *LIPH*), which produces 2-acyl-LPA from phosphatidic acid (PA). The 2-acyl-LPA activates LPAR6, which is also expressed in the epithelial cells. LPAR6 activates ADAM17 which induces ectodomain shedding of TGF-α, leading to transactivation of EGFR, resulting in a proper hair follicle development. *LPAR6* mutations cause deficient LPAR6 enzyme activity, resulting in the defective activation of ADAM17 and loss of LIPH-LPA-LPAR6 signaling [23].

The mutation within the *LPAR6* gene in Cornish Rex leads to a truncation of a protein product prior to the third transmembrane domain. Hair root bulb mRNA derived from a Cornish Rex cat was genotyped and the RNA transcript with the deletion was confirmed [6]. The hair of Cornish Rex is very short, lacks guard and various awn hairs, and falls in Marcel Waves. The Cornish Rex breed originates from a litter in Cornwall, England, in 1950 [24,25]. One of the male kittens had an unusual, finely curly coat. The owner then systematically crossed him and his offspring either with rex- or wild-type domestic cats. The number of wild type and rex phenotypes was consistent with the ratio expected from crosses, where rex phenotype is due to a single fully penetrant recessive mutation [24,25]. Gandolfi et al. (2013) genotyped 12 Cornish Rex cats using a 63k SNP array and identified genomic selection signature signals. They identified a consensus block of homozygosity that spans approximately 3 Mb and includes *LPAR6* gene with Cornish Rex mutation, which is fixed in Cornish Rex and absent in all straight-haired cats they analyzed [6].

In addition to the rexoid coat, Cornish Rex cats also have a characteristic head, ear shape and body type. The analysis of selection signatures in the Cornish Rex genome, focusing on the region harboring the rexoid locus (*LPAR6*), revealed several regions under selective pressure; however, no recognized candidate genes were found [6]. According to a 1965 article in the CFA Year Book, written by one of the early Cornish Rex breeders, Helen Weiss [26], Cornish Rex cats were crossbred with Domestic, Siamese, Burmese and Oriental Shorthair cats. The breed standard of the Cornish Rex was established in 1967, and has been revised several times. Over time, the importance of characteristics other than the coat, such as the shape and size of the head, eyes and ears, as well as body structure, has increased.

In cats, the extent of linkage disequilibrium (LD) was estimated using custom SNP assay containing 1536 SNPs. The mean LD across cat breeds was 96 Kb, and ranged from 17 kb (Siberian breed) to 380 kb (Burmese breed). LD in the Cornish Rex was estimated to be 63 Kb [27]. Later, the genome-wide LD estimation based on the 63k DNA array was in overall agreement with the previously reported estimates [28]. The feline genotyping array containing 63K variants was based on the improved initial [29] feline genome assembly and SNPs across different cat breeds, including Cornish Rex (Abyssinian, American Shorthair, Cornish Rex, European Burmese, Persian, Siamese, Ragdoll and African wildcat) [30]. The availability of feline SNP array shifted the mapping of traits from pedigree-based linkage studies to GWAS for both Mendelian and polygenic traits in cats [31,32,33]. However, genome-wide association studies (GWAS) are not the optimal approach to identify genomic regions contributing to phenotypes in a recessive mode of inheritance [6]. Genomic footprints left by selection are used to identify loci subjected to selection [34]. It has been estimated that at least 0.1–0.7% of the autosomal genome in cat is potentially under selection. Typically, in different cat breeds, between 2 and 12 regions under selection were identified. Breed-specific analyses revealed significant differences in the number and size of candidate regions, influenced by factors such as breed ancestry, population size, the age of breed-defining traits, the mode of inheritance of traits, and breeding practices. For instance, Eastern breeds showed larger and more numerous candidate regions compared to Western breeds, reflecting their distinct breeding histories and the traits selected [35].

In the current study, we genotyped Cornish Rex cats, cats of other breeds, and F1 hybrids between Cornish Rex and domestic cats for the deletion in the *LPAR6* gene associated with the rex phenotype and deletion in the *ALX1* gene, a candidate for brachycephaly. Our study sought to uncover breed-specific selection signatures that could explain phenotypes and characteristic for Cornish Rex cats using runs of homozygosity (ROH) island analysis and integrated haplotype score (iHS) test.

## 2. Materials and Methods

### 2.1. Ethics Statement

Buccal swab samples were obtained by cat owners. No treatments or other procedures with animals were performed that would demand ethical protocols according to Directive 2010/63/EU (2010).

### 2.2. Sample and Data Collection

We collected buccal swab samples from 40 cats (*Felis catus*) of different breeds: Cornish Rex (20), Devon Rex (4), Selkirk Rex (4), Sphynx (1), Don Sphynx (4), domestic cat (3) and F1 hybrid offspring of Cornish Rex and domestic cat (4). Photos of Cornish Rex and domestic cat crosses were obtained from the breeder (Piupaws; http://www.piupaws.net/eparexit_en.html, accessed on 10 January 2024).

### 2.3. DNA Extraction, Fragment Analysis and Sanger Sequencing

Genomic DNA was extracted using E.Z.N.A.^®^ DNA Kit (Omega Bio-Tek, Norcross, GA, USA) according to manufacturer’s recommendations. The Primers for sequencing the region containing *LPAR6* mutation (F-TAATCTAACAGGCTGGCTTCA, R-GCAGGCTTCTGAGGCATTGTT) were obtained from the previously published study by Gandolfi et al. (2013) [6]. Sequences were analyzed and aligned using Mega 11 software [36]. Primers for fragment analysis of the *ALX1* gene region coding homeobox domain with potential deletion of four amino acids (F-AAATCATTAACAGACTGCTTTCCTGA, R-ATGGTTCTAGTCTTTAGTGAGAGGATCA-Fam) were published by Lyons et al. (2016) [37]. The targeted region was amplified using PCR and fragment analysis was performed using ABI3500 gene analyzer.

### 2.4. Remapping of Feline SNP Array Coordinates to F.catus_Fca126_mat1.0

We used publicly available SNP array data (Illumina Infinium iSelect 63K Cat DNA Array) from the study of Gandolfi et al. (2018) containing 11 Cornish Rex samples and 2067 cats of various breeds [28]. The array variants were mapped to cat genome assembly Felis_catus_8.0, and we determined the coordinates of each variant in F.catus_Fca126_mat1.0 [38] genome assembly. For each SNP, 100 bp upstream and 100 bp downstream sequences from the Felis_catus_8.0 were obtained. Then, each 200 bp sequence was aligned to F.catus_Fca126_mat1.0 genome sequence. We used UCSC Blat program [39] to generate alignments with following settings: minimum of 11 bp of matching sequence to initiate an alignment (tileSize = 11) and at least 90% matching bases required (minIdentity = 90), the number of tile matches was 2 (minMatch = 2), the minimum score was 30 (minScore = 30), and the size of the maximum gap between tiles in a clump was 2 (maxGap = 2). The best matches were selected to determine the coordinates of each marker in the new genome assembly.

### 2.5. Identification of Selection Signatures

After SNP remapping to the new genome assembly, we used SNP & Variation Suite v8.10.0 (Golden Helix, Inc., Bozeman, MT, USA, www.goldenhelix.com, accessed on 10 January 2024) software for quality control and ROH island detection based on SNP array. SNPs located on the sex chromosomes and SNPs with a call rate lower than 90% were removed. We used data for 11 Cornish Rex cats, performed PCA analysis, and estimated pairwise IBD for analyzed samples.

We identified runs of homozygosity (ROH) islands in the Cornish Rex population that indicate breed-specific selection signatures. The minimum length of ROH was set to 500 kb, the minimum number of homozygous SNPs within a run was set to 25, and a maximum of five SNPs with missing genotype and no heterozygous SNPs were allowed within a run. ROH islands defined as regions of the genome where individuals share ROH were identified.

Chromosome-wise haplotype phasing was performed with Beagle software version 5.4 [40] with default settings using SNP array dataset containing genomes of 11 Cornish Rex and 2067 cats of various breeds. The ancestral allele was inferred as the most common allele within all 2078 samples. Genome-wide scans for selection signatures were performed with iHS statistics using rehh package v3.2.2 [41] with the default options. The two-tailed Z-test was applied to detect statistically significant SNPs under selection with unusually extended haplotypes of ancestral (reflected by positive iHS values) or derived alleles (indicated by negative iHS values).

### 2.6. Annotation of Regions under Selection

After identification of selection signatures, we performed annotation of protein-coding genes using NCBI database based on Fca126_mat1.0 genome assembly. For each iHS peak, we obtained all protein-coding genes within 250 kb upstream and 250 kb downstream genomic region. Phenotyping annotation was performed using Gene Ontology (GO), AnimalQTLdb, and UniProt.

## 3. Results

### 3.1. LPAR6 Mutation

**Figure 1:**
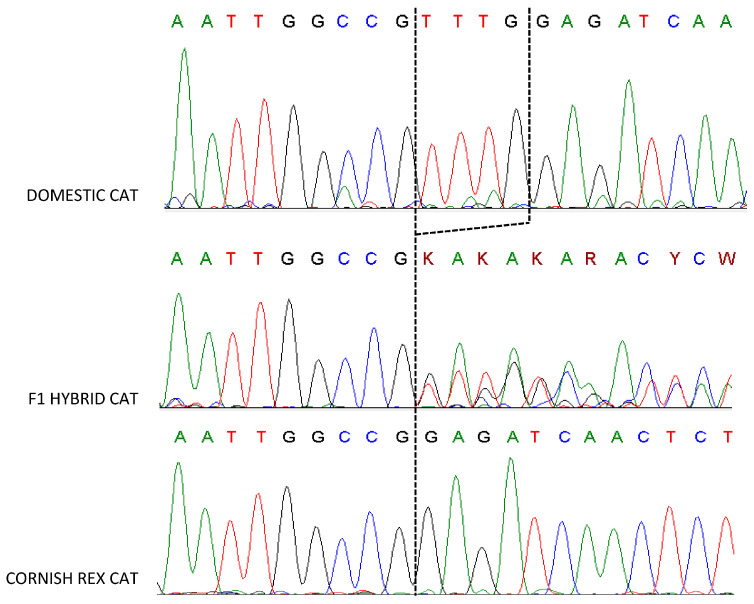
The Cornish Rex sequence has a 4 bp deletion in exon 5 of the *LPAR6* gene. The F1 hybrid (Cornish Rex × Domestic cat) is heterozygous for *LPAR6* mutation. The sequence is unreadable downstream from the deletion, due to two sequencing templates of different length in a heterozygous individual.

We genotyped 40 cats for LPAR6 mutation (c.250_253_delTTTG) (Table 1, Figure 1). All Cornish Rex cats are homozygous for a 4 bp deletion. We also identified a Devon Rex, heterozygous for deletion, although the rex phenotype in Devon Rex breed is associated with KRT71 gene.

### 3.2. ALX1 Genotype

**Figure 2:**
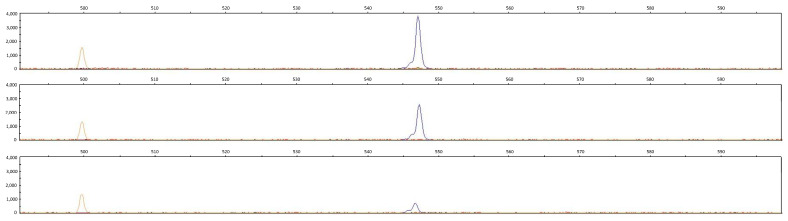
A homozygous state of the homeo box coding region of the ALX1 gene in two Cornish Rex (**upper two rows**) and in one domestic cat (**bottom row**).

The homeobox coding region of the ALX1 locus, which is in Burmese cats bearing a 12 bp deletion, which causes deletion of four amino acids from this domain, was tested for the presence of deletion in Cornish Rex cats. In two genotyped Cornish Rex cats and one domestic cat, we could not see this 12 bp deletion (Figure 2).

### 3.3. Cornish Rex Phenotype

**Figure 3:**
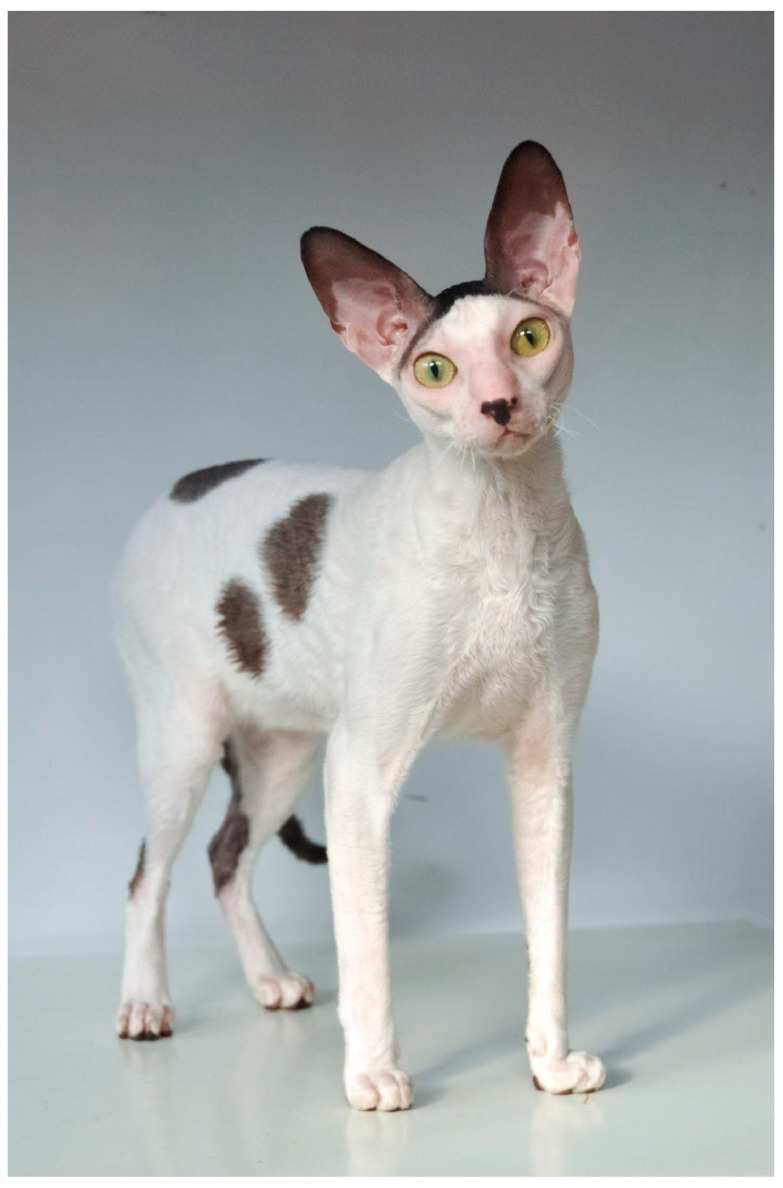
Cornish Rex cat.

The Cornish Rex has several unique phenotypic features in addition to curly coat (Figure 3). They have long and wedge-shaped head. The skull of this cat breed is an egg-like shape. The cat has a strong chin. The nasal line is straight, length of nose is one-third of head. The eyes of this cat breed are large, and have an oval shape. The ears are large, and set high on the head. The Cornish Rex appears to be an active and sociable breed with a low tendency for aggression toward strangers and a curious nature regarding new objects [42].

**Figure 4:**
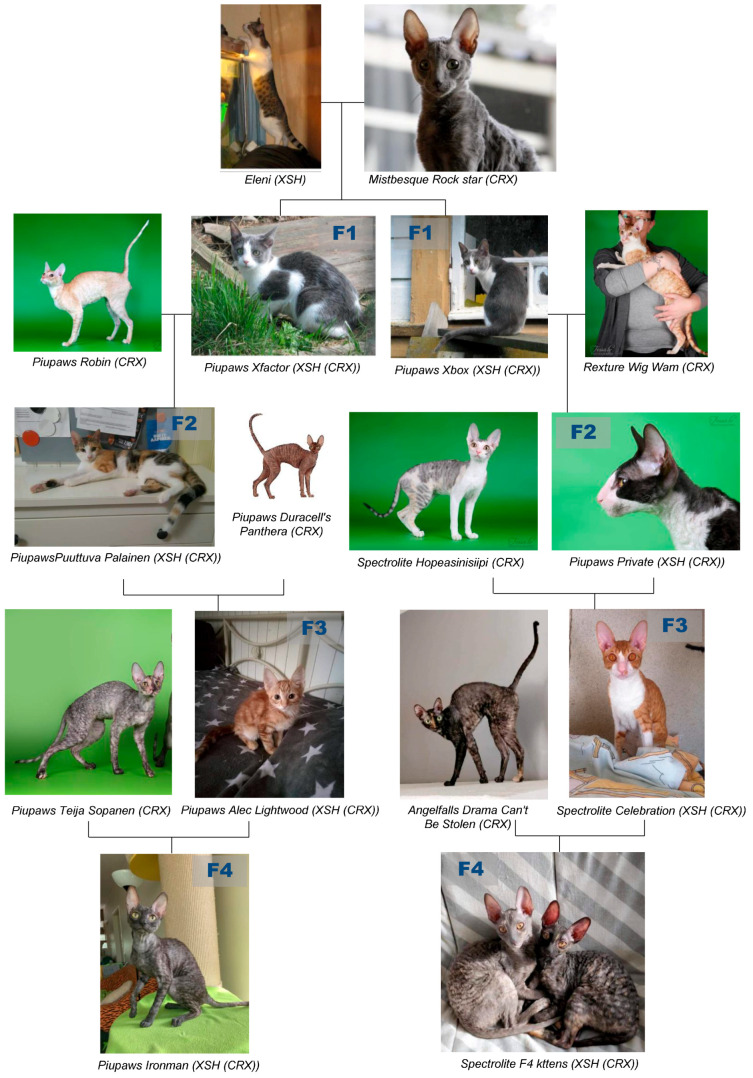
Pedigree of Cornish Rex and domestic cat crosses. The gradual change in head morphology and ear size indicates that increasing proportion of the Cornish Rex genome in cross animals (parents and hybrid generations from F1 to F4) causes specific morphological changes, (CRX—Cornish Rex, XSH—shorthaired hybrid).

In the 1974 article in the CFA Year Book written by Rosemonde Peltz, there is a photograph of Cornish Rex cat (Diamond Lil from cattery OF Fan-T-Cee) with the comment about her expressive head, which is desired in Rex [43]. In the backcross pedigree, the gradual change in head morphology and ear size was identified phenotypically. Increasing proportion of the Cornish Rex genome in cross animals causes specific morphological changes (Figure 4).

### 3.4. Selection Signatures

**Figure 5:**
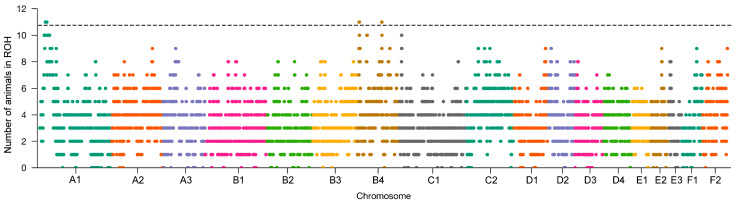
Genome-wide distribution of selection signatures in Cornish Rex cats on chromosomes A1, B4 and C1 identified using ROH islands. The dashed line indicates top 1% of SNPs observed in ROH.

To detect signatures of selection in Cornish Rex cats, we used publicly available SNP array data containing 11 Cornish Rex samples and performed two complementary methods, ROH islands and iHS. Additionally, we obtained SNPs with locus specific divergence (d_i_) values above the 99th percentile in the Cornish Rex from the study of Gandolfi et al. (2013) [6]. On the chromosome B4, Gandolfi et al. (2013) identified 14 markers on the genomic position between 110,006,870 and 110,487,632 bp, where the *LRRIQ1* and *ALX1* genes are situated. We identified the ROH island within the same region, where 7 out of 11 Cornish Rex cats had ROH island. However, ROH islands were identified also on chromosomes A1, B4 and C1 where all animals or 10 of 11 animals had ROH in the same genomic region (Figure 5). ROH island with the *LPAR6* gene was identified on chromosome A1 (Table 2).

**Figure 6:**
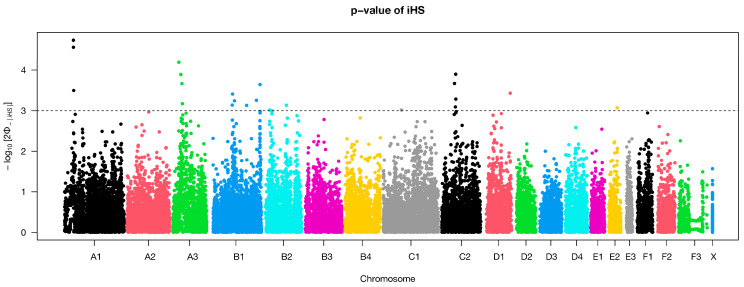
Genome-wide distribution of selection signatures in Cornish Rex cats identified using integrated haplotype score (iHS). Horizontal dashed line marks the significance threshold.

The integrated haplotype score (iHS) statistics was utilized to reveal selection signatures on chromosomes A1, A3, C2, B1 and D1 within Cornish Rex cats (Figure 6). The iHS test based on phased haplotype detected 12 SNPs that defined putative selection signatures in Cornish Rex cats (Table 3). The identified genes within these regions underscore the genetic basis of diverse biological processes. The strong selection signature on chromosome A1 was confirmed by homozygosity and iHS method.

**Figure 7:**
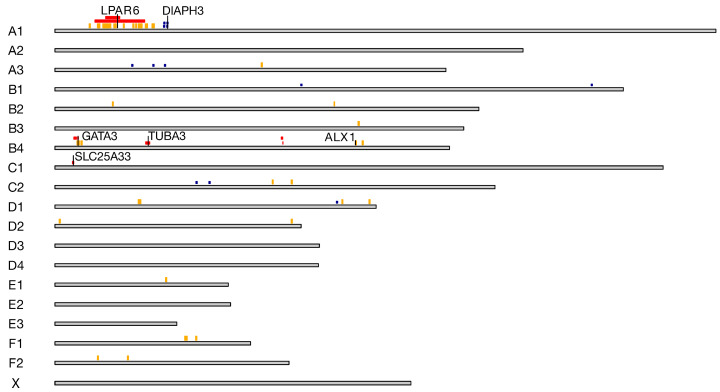
Overlap of selection signatures identified with three different approaches: orange—d_i_ statistics above the 99th percentile, red—ROH islands, blue—selection signatures identified using integrated haplotype score (iHS).

Comparison of locations identified as selection signatures using three different approaches revealed two overlapping regions (Figure 7). On chromosome A1, d_i_ and ROH islands revealed region specific for Cornish Rex where LPAR6 gene is located. On chromosome B4 both approaches overlapped within the region encoding gene *GATA3*.

## 4. Discussion

In the current study, the objective was to genotype Cornish Rex cats for an already known variant in *LPAR6* gene to confirm the mutation and to identify selection signatures based on feline 63K SNP Array. Our genotyping results confirmed the homozygous 4 bp deletion in the *LPAR6* gene [6] in Cornish Rex cats. The curly phenotype was not present in F1 Cornish Rex x domestic cat crosses, but reemerged in the F2 back cross animals, underscoring the recessive nature of this trait. Increasing proportion of the Cornish Rex genome in cross animals causes specific morphological changes, therefore, the presence of other homozygous blocks in the genome, in addition to the one harboring the rexoid locus, is expected.

Previous studies have identified regions under selection in Cornish Rex [6], and we further explored these findings by employing two different complimentary approaches (ROH islands and iHS) to reveal regions under selection in Cornish Rex. We identified ROH island containing *LPAR6* gene on chromosome A1. In the same ROH island, there is also the gene FRAS1 Related Extracellular Matrix 2 (*FREM2*), which plays a critical role during the development of eyelids and the anterior segment of the eyeballs in mice [44], and may play a role in the craniofacial morphology. There is also serotonin receptor gene 2A (*HTR2A*) in the same ROH island. Gene *HTR2A* was associated with aggression and irritability in humans [45,46], and may be associated with Cornish Rex breed temperament, which has according to the study on cat behavior, low tendency for aggression [42]. Gene Myosin IA (*MYO1A)* is located on ROH island on chromosome B4, and is expressed in the inner ear and is a candidate gene for hearing loss in human [47].

The selection signature in Cornish Rex was previously identified in the genomic region harboring the gene *ALX1* on chromosome B4. The deletion (12 bp) in the *ALX1* gene was found to cause craniofacial malformations in Burmese cats [37]. In the description of the development of the Cornish Rex breed, Burmese cats were also listed as a breed that was crossed with Cornish Rex. Therefore, we genotyped the Cornish Rex samples for *ALX1* deletion, but the deletion was not detected in Cornish Rex. We identified the *LPAR6* deletion in pedigree Devon Rex (in heterozygous state), although causal variant for curly coat in this breed is located in *KRT71* gene. The presence of *LPAR6* deletion in Devon Rex indicates crosses between Devon and Cornish Rex in the early stage of breed development.

Further insights were gained from the iHS analysis, identifying additional regions under selection across chromosomes, including A1, A3, C2, B1 and D1. Genes *DIAPH3*, *TDRD3* and *PCDH9* are located within a selection signature on chromosome A1. The *DIAPH3* gene has been linked to body size in equines, with reports of copy number variants in donkeys, ponies, and horses of small stature [48]. In mice, genomic region harboring genes *Diap3*, *Pcdh17*, *Pcdh20* and *Tdrd3* was associated with the length of bones (humerus, ulna, femur, tibia) [49]. The Cornish Rex is a small-to-medium sized breed, and in the breed standard, 30 out of 100 points are assigned to body traits, with 10 of them to the torso, which is described as long and slender. This may be reflected in the selection signature, detected in our analysis.

Gene *pcdh9* was associated with head length in catfish [50]. In catfish, head size affecting genetic loci were mapped to regions with candidate genes involved in bone development. Moreover, comparative analysis indicates that homologs of candidate genes involved in skull morphology are evolutionary conserved ranging from amphibian to mammalian species [50]. The development of vertebrate head is a complex process relying on precise control of several regulatory pathways. The research on facial and cranial development relies on animal models, such as frog, zebrafish, chicken and mouse. Genes that were associated with craniofacial conditions and secondarily in an animal model (zebrafish, mouse) are *CAPZB*, *POLR1A*, *ENDRA*, *FGFR2*, *FOE1*, *GRHL3*, *EIF4A3*, *HCFC1*, *RIT1*, *COLEC11* and *MASP1* [51].

Both approaches, iHS and ROH based identification of selection signatures have revealed significant selection signatures on chromosome A1. ROH islands are useful for detecting strong, recent selection signatures with notable inbreeding or genetic drift. Genomic region with recessive mutation *LPAR6* became homozygous due to selection pressure in Cornish Rex, and we were able to identify this region using ROH islands. The iHS method is useful for identifying alleles under ongoing selection that have not reached fixation in the population.

In the current study, we used a relatively low number of SNP array genotyped samples for genome-wide scans of selection signatures. Given the complex genetic architecture of feline traits, and the diversity within cat breeds, a larger sample size could enhance the robustness of our findings. Based on the findings from this study, future research directions hold prospects for enhancing our understanding of the genetic mechanisms of breed-specific traits in the Cornish Rex and other cat breeds by including a larger number of samples and whole-genome sequencing. Moreover, integrating genomic data with detailed phenotypic records can contribute to identification of associations between genetic variations and their phenotypic effects.

## 5. Conclusions

Genotyping of Cornish Rex and cats from other breeds, including the domestic cat, confirmed the presence of homozygous 4 bp deletion in *LPAR6* gene in Cornish Rex cats as a causal mutation for curly hair. In the backcross pedigree, the gradual change in head morphology and ear size was identified phenotypically, indicating that increasing proportion of the Cornish Rex genome in cross animals causes specific morphological changes.

Bioinformatic analysis of 63K Cat DNA Array data revealed selection signature on the FCA B4 in the region, where *ALX1* gene, which was identified as a causal locus for brachycephaly in Burmese cats, is situated. Our analysis could not confirm that deletion in *ALX1* gene would be present in Cornish Rex breed. However, we identified genomic region under selection containing *LPAR6* deletion. We conclude that genome scans of signatures of selection can be used for identification of genomic regions contributing to distinctive morphology, including a distinctive head, ear shape, and body type characteristic for the breed.

Investigating the genetic foundations underlying breed-specific characteristics, such as those observed in the Cornish Rex, provides not only a deeper understanding of the breed’s history, but also contributes significantly to the broader fields of genetics, and the conservation of genetic diversity within domestic species, which is essential for the health and viability of domesticated populations.

## Figures and Tables

**Table 1:** Genotypes of LPAR6 in 40 cats.

			Sequence			
			5′UTR-194	5′UTR-72	E5 63	E5 250-253
Breed	Coat Phenotype	N	G/A	C/T	C/T	TTTG/del
Cornish Rex	Curly	20	G	C	C	del
Devon Rex	Curly	4	G	C/T	C	TTTG/del
Selkirk Rex	Curly	4	G	C/T	C/T	TTTG
Sphynx	Hairless	1	G	C	C	TTTG
Don Sphynx	Hairless	4	G	C	C	TTTG
Domestic cat	Straight hair	3	G	T	C	TTTG
Cornish Rex × Domestic cat F1	Straight hair	4	G	C	C	TTTG/del
All		40				

**Table 2:** The list of genomic regions of ROH islands identified in Cornish Rex.

Genomic Position	Number of Animals in ROH	Protein Coding Genes in ROH Islands
A1: 18,541,422–23,597,569	11	*FOXO1*, *TRNAR-UCG*, *MRPS31*, *SLC25A15*, *THSD1*, *VPS36*, *CKAP2*, *NEK3*, *NEK5*, *ALG11*, *ATP7B*, *CCDC70*, *TMEM272*, *DHRS12*, *WDFY2*, *INTS6*, *SERPINE3*, *FAM124A*, *CA1H13orf42*, *RNASEH2B*, *DLEU7*, *KCNRG*, *TRIM13*, *SPRYD7*, *KPNA3*, *EBPL*, *ARL11*, *RCBTB1*, *PHF11*, *SETDB2*, *CAB39L*, *CDADC1*, *MLNR*, *FNDC3A*, *CYSLTR2*, *LOC109497974*, *RCBTB2*, *RB1*, *LPAR6*, *ITM2B*, *MED4*, *NUDT15*, *SUCLA2*
B4: 7,564,954–8,554,345	11	*TAF3*, *GATA3*
B4: 82,833,573–83,377,506	11	*STAT2*, *APOF*, *TIMELESS*, *MIP*, *SPRYD4*, *GLS2*, *RBMS2*, *BAZ2A*, *ATP5F1B*, *PTGES3*, *NACA*, *PRIM1*, *HSD17B6*, *SDR9C7*, *RDH16*, *GPR182*, *ZBTB39*, *TAC3*
A1: 14,659,146–32,635,718	10	*DCLK1*, *SOHLH2*, *CCDC169*, *SPART*, *CCNA1*, *SERTM1*, *RFXAP*, *SMAD9*, *ALG5*, *EXOSC8*, *SUPT20H*, *POSTN*, *TRPC4*, *UFM1*, *FREM2*, *STOML3*, *PROSER1*, *NHLRC3*, *LHFPL6*, *COG6*, *FOXO1*, *TRNAR-UCG*, *MRPS31*, *SLC25A15*, *THSD1*, *VPS36*, *CKAP2*, *NEK3*, *NEK5*, *ALG11*, *ATP7B*, *CCDC70*, *TMEM272*, *DHRS12*, *WDFY2*, *INTS6*, *SERPINE3*, *FAM124A*, *CA1H13orf42*, *RNASEH2B*, *DLEU7*, *KCNRG*, *TRIM13*, *SPRYD7*, *KPNA3*, *EBPL*, *ARL11*, *RCBTB1*, *PHF11*, *SETDB2*, *CAB39L*, *CDADC1*, *MLNR*, *FNDC3A*, *CYSLTR2*, *RCBTB2*, *RB1*, *LPAR6*, *ITM2B*, *MED4*, *NUDT15*, *SUCLA2*, *HTR2A*, *ESD*, *LRCH1*, *RUBCNL*, *LRRC63*, *LCP1*, *CPB2*, *ZC3H13*, *CBY2*, *SIAH3*, *ERICH6B*, *COG3*, *SLC25A30*, *TPT1*, *GTF2F2*, *KCTD4*, *GPALPP1*, *NUFIP1*, *TRNAE-UUC*, *TSC22D1*, *SERP2*, *SMIM2*, *LACC1*, *CCDC122*, *ENOX1*, *DNAJC15*, *EPSTI1*, *FAM216B*, *TNFSF11*, *AKAP11*, *DGKH*, *VWA8*, *RGCC*, *NAA16*, *MTRF1*, *KBTBD7*, *WBP4*, *ELF1*, *TRNAE-UUC*, *SUGT1*, *CNMD*, *PCDH8*, *OLFM4*
B4: 8,604,768–8,903,308	10	*LOC123386437*, *LOC123386806*
B4: 33,388,421–34,885,172	10	*USP18*, *TUBA8*, *PEX26*, *MICAL3*, *BID*, *BCL2L13*
B4: 83,404,047–83,486,525	10	*MYO1A*, *NAB2*, *STAT6*, *LRP1*
C1: 6,410,172–6,908,245	10	*H6PD*, *SPSB1*, *SLC25A33*, *TMEM201*, *PIK3CD*, *CLSTN1*

**Table 3:** Putative signatures of selection identified with iHS method within Cornish Rex cats.

Marker	Chromosome	Position (bp)	iHS	*p*-Value	Protein Coding
chrA1.45401576	A1	40,003,327	−4.28	4.74	*DIAPH3*, *TDRD3*, *PCDH9*
chrA1.45490019	A1	40,077,392	4.19	4.56	*TDRD3*, *PCDH9*
chrA3.333763	A3	28,335,268	−4.00	4.19	*SNPH*, *RAD21L1*, *SDCBP2*
chrC2.67784789	C2	56,640,227	−3.83	3.90	*CD47*, *IFT57*, *HHLA2*, *MYH15*, *DZIP3*, *MORC1*, *NECTIN3*, *CD96*, *PHLDB2*
chrA3.41536320	A3	36,072,528	−3.83	3.89	*SNAP25*
chrUn33.3409815	C2	51,884,775	−3.70	3.67	*ZPLD1*, *ALCAM*, *CBLB*
chrA3.46750993	A3	40,332,715	−3.70	3.67	*MACROD2*, *KIF16B*, *SEL1L2*
chrB1.218769158	B1	196,651,922	−3.69	3.64	*KCNIP4*, *SLIT2*, *LDB2*, *CLNK*, *SLC2A9*, *STK32B*
chrA1.46779148	A1	41,152,849	3.60	3.50	*DIAPH3*, *TDRD3*
chrA1.46838659	A1	41,215,975	3.60	3.50	*DIAPH3*, *TDRD3*
chrUn18.8527193	D1	103,331,049	−3.56	3.43	*CNTF*, *ZFP91*, *GLYAT*, *GLYATL2*
chrB1.111674326	B1	90,268,797	−3.55	3.41	*ELF2*, *SCLT1*

## Data Availability

Data are contained within the article.
